# Supplementary material for: Learning curve and surgical outcome of robotic assisted colorectal surgery with ERAS program
Source: Sci Rep. 2022 Nov 29;12:20566. doi: 10.1038/s41598-022-24665-w (PMC9709162; doi:10.1038/s41598-022-24665-w)
Supplement: Supplementary file 2 — Supplementary Information 2. [file 41598_2022_24665_MOESM2_ESM.zip › RAL Raw data-Ñ[▒K/139/OP N_ñw╝╨░O▒Kñσ.pdf]

W83 / W83

臺中榮民總醫院
手術室護理記錄

[redacted]

基本資料

- 室別： 開刀房-17室，手術科別： CRS，手術類別： 常規，日期： 2022/01/05
- 進入時間： 0755 ； 麻醉開始時間： 0810 ； Time Out時間： 0855 ； 劃刀時間： 0856 ； 手術結束時間： 1415 送出時間： 1440 ；
- 麻醉方式： GE

  診斷：rectal cancer s/p SCRT

  手術名稱：Robot Assisted Low Anterior Resection

  手術局部用藥：NIL

  術前預防性抗生素：
  - 抗生素名稱一： Veterin 1GM IVD/由麻醉護理師給
  - 時間： 0850
  - 上次簽章: 何采穎

  , 追加預防性抗生素：
  - 抗生素名稱一： Veterin 1GM IVD/由麻醉護理師給
  - 時間： 1315
  - 上次簽章: 何采穎
- 手術醫師： 林俊余，楊曙榮，陳威遠，陳凱祥，陳怡豪
- 麻醉醫師： VS 陳永銘
- 刷手護士A ： 黃宸盈，時間： 0755，接替刷手一： 歐真勤，時間： 1200，接替刷手二： 黃宸盈，時間： 1230

  流動護士A ： 何采穎，時間： 0755，接替流動一： 歐真勤，時間： 1130，接替流動二： 何采穎，時間： 1200

皮膚狀況

A-V Shunt： 無

術前： 完整

記錄者： 上次簽章: 何采穎

術後： 完整

記錄者： 何采穎

手術準備

手術臥式 單一手術臥式

-
  - 手術臥式： 截石臥式
  - 使用附件： 溫毯 40 ℃，腳架，其它： 海棉盒
  - 皮膚準備： 10%Aq Betadine，Hibiscrub，2% Chlorhexidine
  - 電燒： 使用電燒: 電極片位置： 其它： 迴路電極墊，記錄者： 上次簽章: 何采穎

止血帶T： 無

植入物

- STAPLER/45 4.3mm/rectal 釘子四枝

, 記錄者: 上次簽章: 何采穎

標本送檢

- 細菌培養 無
- 細胞學 無
- 冰凍切片 無
- 病理標本

  組織名稱一：DISTAL CUT END

  ,

  組織名稱二：IMA root

  ,

組織名稱三：rectum

- 截肢 無

標本交付 標本： 3 袋，標本： 0 盒，標本： 0 瓶，細胞學： 0 管，Culture Tube： 0 管，被交付醫師： Fellow 楊曙榮，

- 記錄者： 上次簽章: 何采穎
- 時間： 1350

傷口術後數料

傷口數料： 傷口數料： 無

- 請於圖上標示部位：

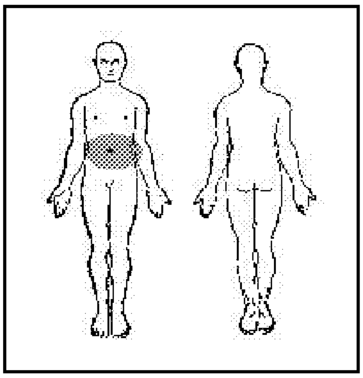

- 傷口數料： MEPORE

導管

導管	天數	導管資訊	插入日期	插入時間	更換日期	拔除日期	拔除時間	拔除原因	預計拔除/更換	預計拔除/更換日期
A-line (1)	1	號碼:20 Ga; 部位:左手;	2022/01/05	0830						
Foley(1)	1	號碼:16 Fr.; 部位:膀胱; 註記:ballon 10 ml;	2022/01/05	0835						
Sump NG Tube(1)	1	號碼:16 Fr.; 部位:胃; 註記:55cm;	2022/01/05	0825						
Hemo Vac(1)	1	號碼:15 Fr.; 部位:骨盆腔;	2022/01/05	1340						
Double Lumen CVC(1)	1	號碼:8 Fr; 深度:14cm; 部位:jugular (右); 象限:RU;	2022/01/05	0820						

用物計數

- 手術中出血量： 100 c.c.
- 手術中尿量： 250 c.c.
-
  - 其它： 無

用物計數： 多切口同時縫合

- 1.器械： 正確，2.縫針： 正確，3.刀片： 正確，4.紗布： 正確，5.腹部墊： 未使用，6.紗球： 未使用，7.PEANUTS ： 未使用，8.紗條： 正確，9.絲帶： 未使用，10.針頭： 未使用，11.Vessel loop： 未使用，12.Boots： 未使用，13.Jelco： 未使用，14.棉條： 未使用，
  - 第一次：
    - 刷手護士 ： 黃宸盈
    - 流動護士： 上次簽章: 何采穎
    - 時間： 1340

  ,
  - 第二次：
    - 刷手護士 ： 黃宸盈
    - 流動護士： 上次簽章: 何采穎
    - 時間： 1350

  ,
  - 第三次：

- 刷手護士 ： 黃宸盈
- 流動護士： 上次簽章: 何采穎
- 時間： 1400

備註

備註： davinci 0度 XI scopy:No.1
davinci 30度 XI scopy:No.1

- 記錄者： 上次簽章: 何采穎
- 時間： 0830

填寫護理人員 何采穎

正式摘要

W83 / W83

doctorRank:B
